# Clavicular tunnel widening in chronic acromioclavicular joint instabilities after primary versus revision arthroscopically‐assisted acromio‐ and coracoclavicular stabilization

**DOI:** 10.1002/jeo2.12114

**Published:** 2024-09-17

**Authors:** Philipp Vetter, Frederik Bellmann, Larissa Eckl, Asimina Lazaridou, Markus Scheibel

**Affiliations:** ^1^ Department of Traumatology University Hospital Zurich Zurich Switzerland; ^2^ Department of Shoulder and Elbow Surgery Schulthess Clinic Zurich Switzerland; ^3^ Department of Shoulder and Elbow Surgery, Center for Muskuloskeletal Surgery Charité Universitaetsmedizin Berlin Germany

**Keywords:** acromioclavicular joint instability, arthroscopically‐assisted, TightRope, tunnel widening

## Abstract

**Purpose:**

To evaluate joint reduction (loss of reduction [LOR]; dynamic posterior translation [DPT]) and clavicular tunnel widening (cTW) in patients treated with arthroscopically‐assisted acromioclavicular joint (ACJ) stabilization after previously failed nonoperative versus surgical treatment.

**Methods:**

Patients undergoing arthroscopically‐assisted ACJ stabilization (bidirectional tendon allograft with a low‐profile TightRope) after previously failed nonoperative versus surgical treatment were included retrospectively. Bilateral anteroposterior stress views served for evaluating LOR (side‐comparative coracoclavicular distance [CCD]) and cTW at a 6‐weeks‐ and 6‐months‐follow‐up (FU) and for evaluating the filling ratio (FR, vertical device insertion depth relative to clavicle height) at the 6‐weeks‐FU. Postoperative DPT was assessed on Alexander's views.

**Results:**

Twenty‐seven patients (20 male, mean age 46.1 ± 14.8 years) were included (prior treatment: nonoperative: *n* = 15; surgical: *n* = 12). There were no differences in LOR, DPT or cTW between groups postoperatively. Initial CCD‐symmetry at the 6‐weeks‐FU (CCD: −0.1 mm [95% confidence interval, CI, −2 to 1.4 mm]) was followed by LOR at the 6‐months‐FU (CCD: −3.5 mm [95% CI, −5.2 to −1.9 mm]; *p* < 0.001). cTW increased towards the inferior cortex, compared to the superior cortex and the intermediate level (*p* < 0.001, respectively). cTW at the inferior cortex was associated with more LOR (*r* = −0.449; *p* = 0.024) and DPT (*r* = 0.421; *p* = 0.036), dependent on a smaller FR (*r* = −0.430; *p* = 0.032).

**Conclusion:**

Patients undergoing arthroscopically‐assisted ACJ stabilization for chronic bidirectional ACJ instabilities showed comparable radiologic results after previous nonoperative versus surgical treatment. cTW increased towards the inferior cortex and was associated with recurrent vertical and horizontal instability, related to a smaller FR. More research into reduced cTW, for example, by a more filling device, should be performed.

**Level of Evidence:**

Level III, retrospective comparative study.

**Abbreviations**
AC: acromioclavicular
ACJ: acromioclavicular joint
CC: coracoclavicular
CCD: side‐comparative coracoclavicular distance
cTW: clavicular tunnel widening
DPT: dynamic posterior translation
FR: filling ratio
FU: follow‐up
LOR: loss of reduction
LPTR: low‐profile TighttRope

## INTRODUCTION

Acromioclavicular joint (ACJ) dislocations, common injuries of the shoulder girdle [22], can be classified as chronic when the injury‐to‐treatment interval exceeds 21 days [5, 12, 20]. Surgical intervention in such cases is widely performed arthroscopically‐assisted [5, 13, 16, 17, 28]. Material‐wise, tendon grafts are regularly used [4, 5, 9, 17, 21, 27, 28], compensating for reduced or expired biologic healing potential of the native ligaments [11]. Tendon grafts can be augmented with a synthetic implant to reduce tendon load during the initial healing [25] and are strongly recommended for high‐grade bidirectional ACJ instability to restore acromioclavicular (AC) and coracoclavicular (CC) stability [13, 17, 28].

Revision surgery in the chronic setting after previously failed nonoperative versus surgical treatment can be challenging considering altered scapular kinematics [7, 8], osseous integrity [10, 17, 19, 27] or persistent instability [11].

Postoperatively, recurrent vertical instability by means of loss of reduction (LOR) [4, 9, 10, 18, 21, 27, 33] and recurrent horizontal instability by means of dynamic posterior translation (DPT) [16, 17] as well as clavicular tunnel widening (cTW) [3, 4, 5, 19, 29] may occur.

LOR and DPT can be relevant for cosmesis, pain and function [9, 10, 18, 21, 33], while cTW can compromise stability up to a subsequent fracture [10, 19].

To account for this tunnel‐related complication, tendon loop techniques avoiding drilling in chronic cases have been introduced [2], which have shown promising radiographic results [27].

A previous study on acute ACJ instabilities found that cTW at the singular tunnel for synthetic augmentation is conical in shape, increases towards the inferior cortex [3] and can be associated with LOR.

The aim of our study was to compare the radiologic results of chronic ACJ dislocations treated arthroscopically‐assistted with ACJ stabilization by use of a looped CC and transacromial tendon allograft augmented with a single low‐profile TightRope device (LPTR) (Arthrex, Naples, FL, USA) after previously failed nonoperative versus surgical treatment.

We hypothesized that cases with prior surgical treatment would display worse outcomes than patients with previous nonoperative treatment.

## METHODS

All patients provided written consent for study inclusion. Ethical approval was granted by the local ethics commission of Zurich (BASEC‐Nr. 2021‐00675).

For this retrospective study, our local ACJ instability registry (Schulthess Clinic Zurich) was searched for patients with chronic bidirectional ACJ instability treated with arthroscopically‐assisted CC and AC stabilization by use of a looped CC and transacromial tendon allograft (M. gracilis or semitendinosus), augmented with a single LPTR device (Arthrex, Naples, FL, USA) [2] between 2018 and 2022.

Thirty‐three patients were screened based on the eligibility criteria. A minimum 6‐months‐follow‐up (FU) with radiographic imaging (bilateral anteroposterior stress radiographs [10 kg] at 6 weeks‐ and 6‐months‐FU and bilateral Alexander's views [1] at 6‐weeks‐FU) was set as a requirement. Patients with incomplete data sets (*n* = 5) and individuals with prior contralateral ACJ injuries (dislocations, clavicle fractures) and those with ipsilateral clavicle fractures were excluded (*n* = 1).

A chronic ACJ instability was diagnosed by the combination of patient history, clinical examination and radiographic evaluation. All patients had undergone a prior physiotherapeutic trial over 3−6 months and presented with persistent pain and functional complaints.

The surgical technique has been described before [2, 31]. It is noted that the only created tunnel (transclavicular–transcoracoidal) is utilized for implanting the augmenting synthetic device. The initial drilling involves a 2 mm K‐wire, followed by overdrilling with a cannulated 5.1 mm drill at the superior clavicular cortex to accommodate the seating of the button. Subsequent overdrilling is then conducted using a 3.5 mm drill bit.

Radiographic evaluation was performed in an image archiving system (JiveX; VISUS Health IT) with a measurement accuracy of 0.1 mm. The known diameter (5.1 mm) of the LPTR device served for scaling (corrective factor) of measurements.

Bilateral anteroposterior stress views served for evaluating vertical instability [24] (side‐comparative coracoclavicular distance [CCD]) at preoperatively, 6‐weeks‐FU and 6‐months‐FU. Recurrent vertical instability by means of LOR was defined as an increase in CCD from the 6‐weeks‐FU to the 6‐months‐FU, indicated by negative values. Recurrent horizontal instability by means of DPT was assessed on bilateral Alexander views preoperatively and at the 6‐months‐FU (no/partial/complete) [16].

**Figure 1:**
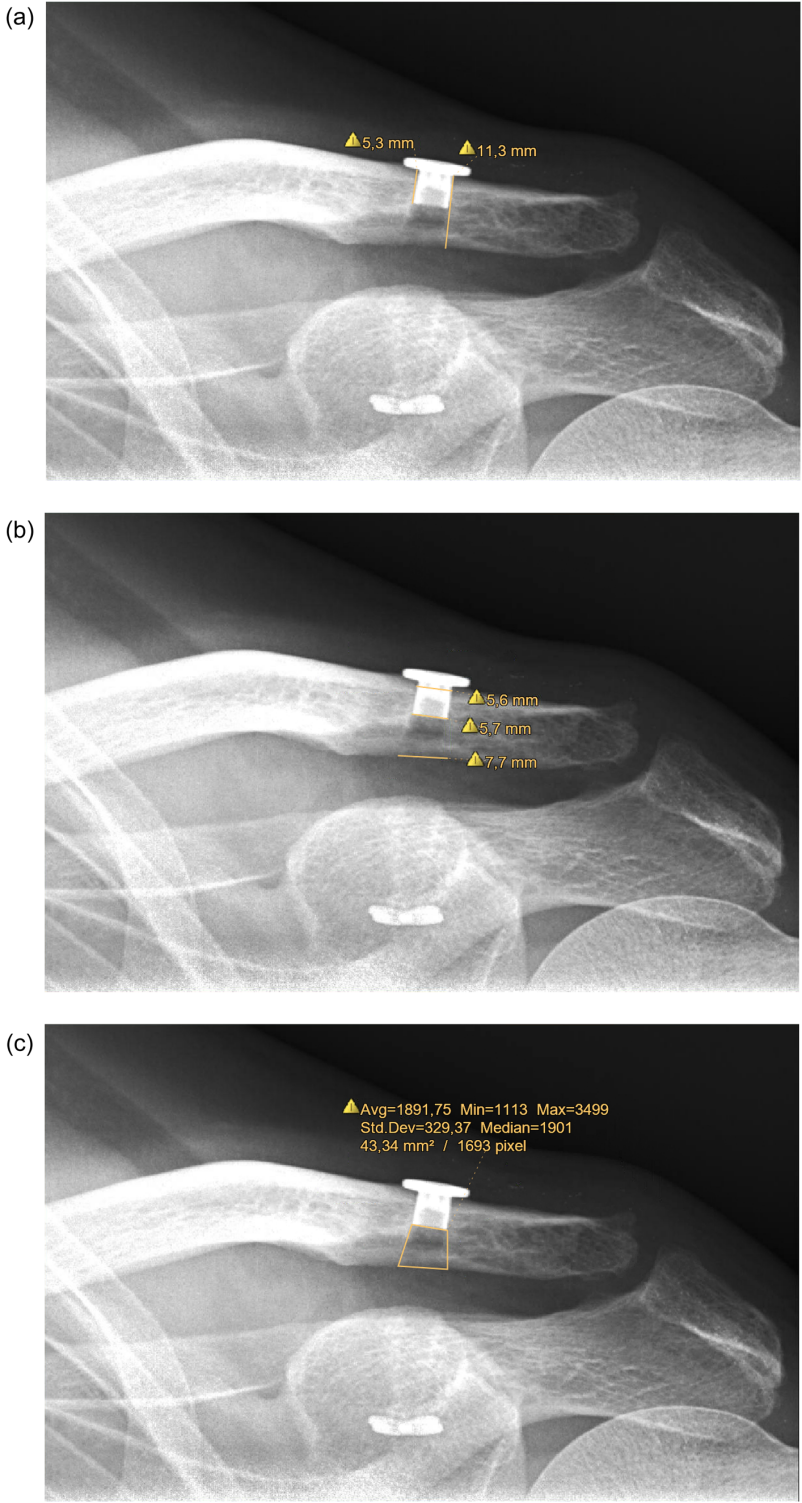
Measurement of parameters around the clavicular drill hole. (a) Measurement of clavicular height and button insertion depth. The filling ratio describes the button insertion depth divided by the clavicular height. (b) Measurement of the tunnel diameter at the superior/inferior cortex and intermediate level. (c) Measurement of the tunnel area below the button.

Parameters around the clavicular drill hole [3] of the LPTR were measured at the 6‐weeks‐FU and the 6‐months‐FU: Clavicular height, button insertion depth, filling ratio (FR), tunnel diameter at superior/inferior cortex and intermediate level, tunnel area below the button, caudal sintering of the top button, osteolysis (Figure 1). FR is defined as the vertical height of the button into the clavicle divided by clavicular height. It was assessed for standardization to account for the vertical osseous proportion exposed to the suture material.

The mediolateral clavicular drill hole position at the 6‐weeks‐FU was measured according to Kraus et al. [17]

### Statistics

Data was analyzed using SPSS 27.0 software (IBM Corp.). Age is reported with a standard deviation. Differences between the primary and revision groups were analyzed by using the Mann−Whitney *U* test after testing for normal distribution according to the Shapiro−Wilk test. Comparison between paired timepoints for the same parameters was performed by applying the Wilcoxon signed‐rank test. The chi‐square test was utilized to test differences in categorical data. Linear regression analysis for continuous variables with normal distribution was performed using Pearson's correlation. Otherwise, a Spearman's rank correlation was used. The level of significance was set at *p* < 0.05.

## RESULTS

### Patient cohort

Twenty‐seven patients (7 female, 20 male, mean age 46.1 ± 14.8 years [range 17−67]) treated with arthroscopically‐assisted CC and AC stabilization by use of a looped CC and transacromial tendon allograft (M. gracilis or semitendinosus) augmented with a single LPTR device (Arthrex, Naples, FL, USA) could be included.

There were 15 patients with prior nonoperative treatment compared to 12 patients with previous surgery (hook plate fixation, *n* = 4; single‐TightRope stabilization, *n* = 2; double‐TightRope stabilization, *n* = 1; single‐TightRope with additional AC stabilization, *n* = 2; single‐TightRope with a free tendon graft, *n* = 1; lateral clavicle resection, *n* = 2). One patient underwent a second hook plate fixation after a failed initial fixation. All patients presented with a clinically and radiographically confirmed bidirectional ACJ instability.

Preoperative characteristics did not differ between groups (Table 1).

**Table 1:** Preoperative characteristics between groups.

	Primary (*n* = 15)	Revision (*n* = 12)	*p* Value
Mean age at (revision) surgery [years]	48.1 ± 16.7 (range, 17−69)	42.4 ± 12.1 (range, 25−63)	0.427
Gender [male/female]	11/4	9/3	1
Injury‐to‐(revision) surgery‐interval [days]	609 (range, 117−3215)	697 (range, 143−2226	0.799
Tendon graft type (semitendinosus/gracilis)	8/7	10/2	0.217
Rockwood type (II/III/V)	0/8/7	2/5/5	0.535

#### Recurrent vertical and horizontal instability

**Figure 2:**
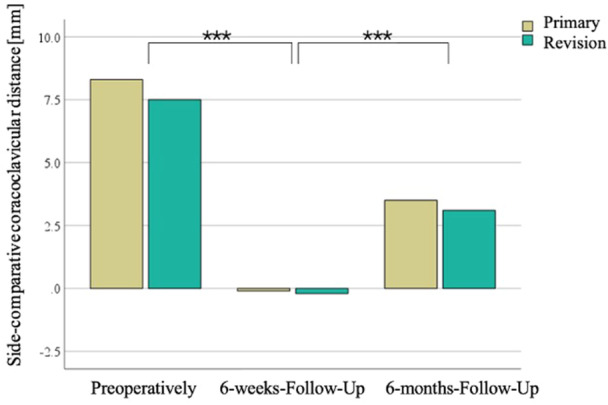
Side‐comparative coracoclavicular distance, between preoperatively, 6‐weeks‐follow‐up and 6‐months‐follow‐up. ***p < 0.001.

There was no preoperative group difference in CCD (primary: CCD: 8.3 mm, 95% CI, 6.1−10.4 mm vs. revision: 7.5 mm, 95% CI, 5.1−9.9 mm; *p* = 0.527) (Figure 2).

Compared to preoperatively (7.9 mm, 95% CI, 6.4−9.4 mm), the CCD at the 6‐weeks‐FU was lowered, displaying a side‐comparative symmetry (−0.1 mm, 95% CI, −2 to 1.4 mm; *p* < 0.001), without group differences (primary: −0.1 mm, 95% CI, −3.6 to 2.5 mm vs. revision: −0.2 mm, 95% CI, −2.5 to 2.0 mm).

However, LOR at the 6‐months‐FU was observed (−3.5 mm, 95% CI, −5.2 to −1.9 mm with CCD: 3.3 mm, 95% CI, 1.8−4.9 mm; *p* < 0.001), again without group differences in LOR (primary: −3.6 mm, 95% CI, −6.5 to −0.9 mm vs. revision: −3.3 mm, 95% CI, −5.5 to −1.2 mm; *p* = 0.769) or absolute CCD (primary: 3.5 mm, 95% CI, 2.2−6.9 mm vs. revision: 3.1 mm, 95% CI, 0.9−5.3 mm; *p* = 0.894) (Figure 2).

Recurrent horizontal instability (DPT) at the 6‐months‐FU was also comparable between groups, being stable in most cases (no/partial/complete—primary: 10/3/2 vs. revision: 9/2/1; *p* = 0.880).

#### cTW

On average, almost half of the clavicle height was filled by the implant device (FR: 0.47 with 95% CI, 0.45−0.49).

There were no significant differences between groups in clavicular height (primary: 10.8 mm, 95% CI, 10.3−11.3 mm vs. revision: 10.7 mm, 95% CI, 9.9−11.5 mm; *p* = 0.943), FR (primary: 0.47, 95% CI, 0.44−0.49 vs. revision: 0.47, 95% CI, 0.44−0.51; *p* = 0.943) or mediolateral clavicular tunnel position, although clavicular tunnel position tended to be more lateral in revision cases (primary: 30.3 mm, 95% CI, 26.8−33.9 mm vs. 25.7 mm, 95% CI, 21.7−29.7 mm; *p* = 0.126).

**Figure 3:**
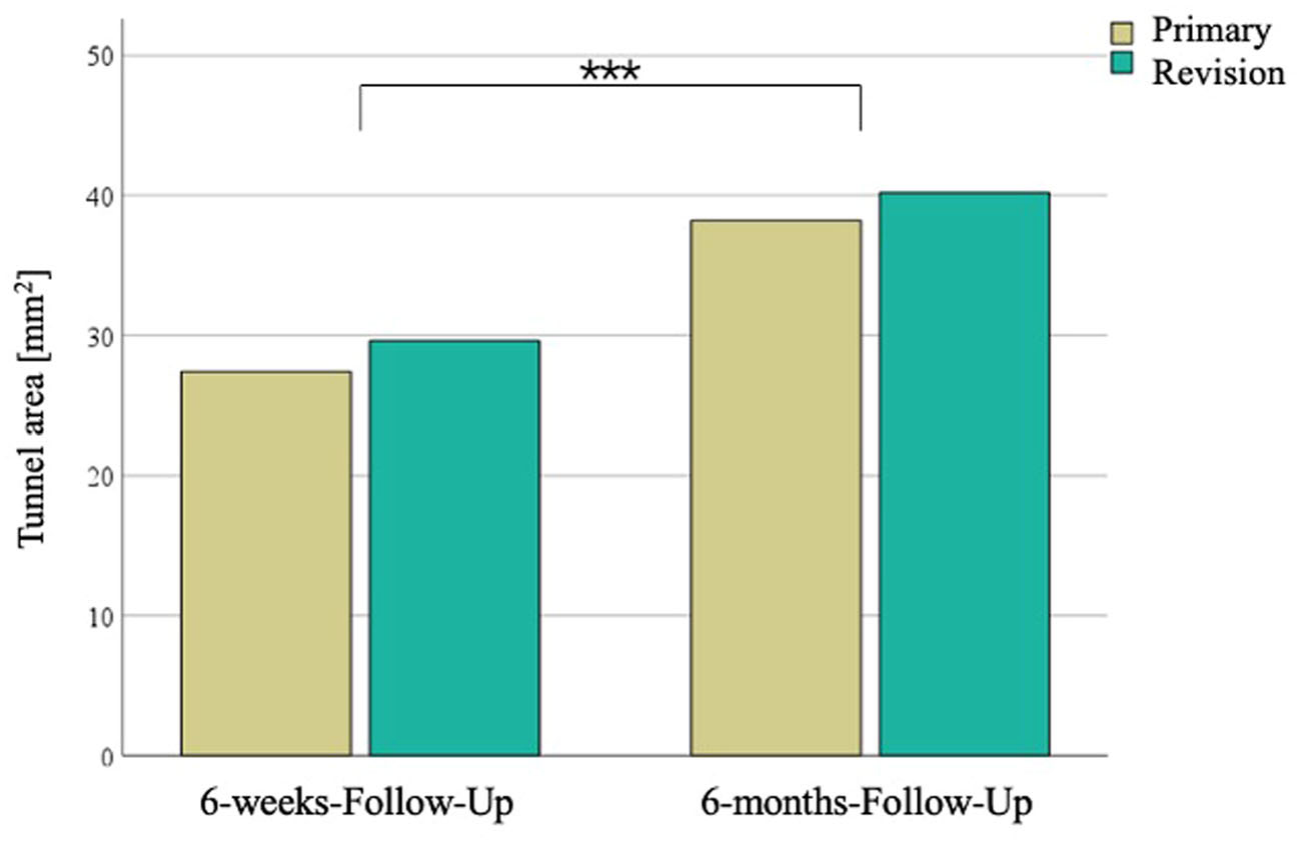
Clavicular tunnel area at the 6‐weeks‐follow‐up and at the 6‐months‐follow‐up. ***p < 0.001.

Clavicular tunnel area increased significantly between the 6‐weeks‐FU and 6‐months‐FU (28.5 mm^2^, 95% CI, 24.2−32.8 mm^2^ vs. 39.1 mm^2^, 95% CI, 34.4−43.8 mm^2^; *p* < 0.001) (Figure 3), but there were again no group differences at the FUs (Table 2).

**Table 2:** Clavicular tunnel measurements between groups.

Parameter	Primary (*n* = 15)	Revision (*n* = 12)	*p* Value
At 6‐weeks‐follow‐up			
Tunnel width superior clavicular level [mm] (95% CI)	5.7 (5.5−5.9)	5.6 (5.4−5.9)	0.437
Tunnel width intermediate clavicular level [mm] (95% CI)	6.0 (5.6−6.3)	5.8 (5.5−6.0)	0.295
Tunnel width inferior clavicular level [mm] (95% CI)	4.8 (4.2−5.5)	4.9 (4.2−5.6)	0.728
Tunnel area [mm^2^] (95% CI)	27.4 (23.2−31.6)	29.6 (21.0−38.1)	0.769
At 6‐months‐follow‐up			
Tunnel width superior clavicular level [mm] (95% CI)	5.9 (5.8−6.1)	6.0 (5.7−6.3)	0.755
Tunnel width intermediate clavicular level [mm] (95% CI)	6.2 (5.9−6.5)	6.2 (5.8−6.5)	0.943
Tunnel width inferior clavicular level [mm] (95% CI)	7.4 (6.7−8.0)	7.3 (6.9−7.8)	0.548
Tunnel area [mm^2^] (95% CI)	38.2 (33.0−43.5)	40.2 (30.8−49.6)	0.867
Differences between 6‐weeks‐follow‐up and 6‐months‐follow‐up			
Tunnel width superior clavicular level [mm] (95% CI)	0.3 (0.1−0.5)	0.4 (0.3−0.6)	0.406
Tunnel width intermediate clavicular level [mm] (95% CI)	0.2 (0−0.5)	0.4 (0.2−0.6)	0.270
Tunnel width inferior clavicular level [mm] (95% CI)	2.8 (2.1−3.5)	2.6 (1.9−3.2)	0.611
Tunnel area [mm^2^] (95% CI)	11.4 (8.2−14.5)	10.6 (6.6−14.7)	0.437

Abbreviation: CI, confidence Interval.

**Figure 4:**
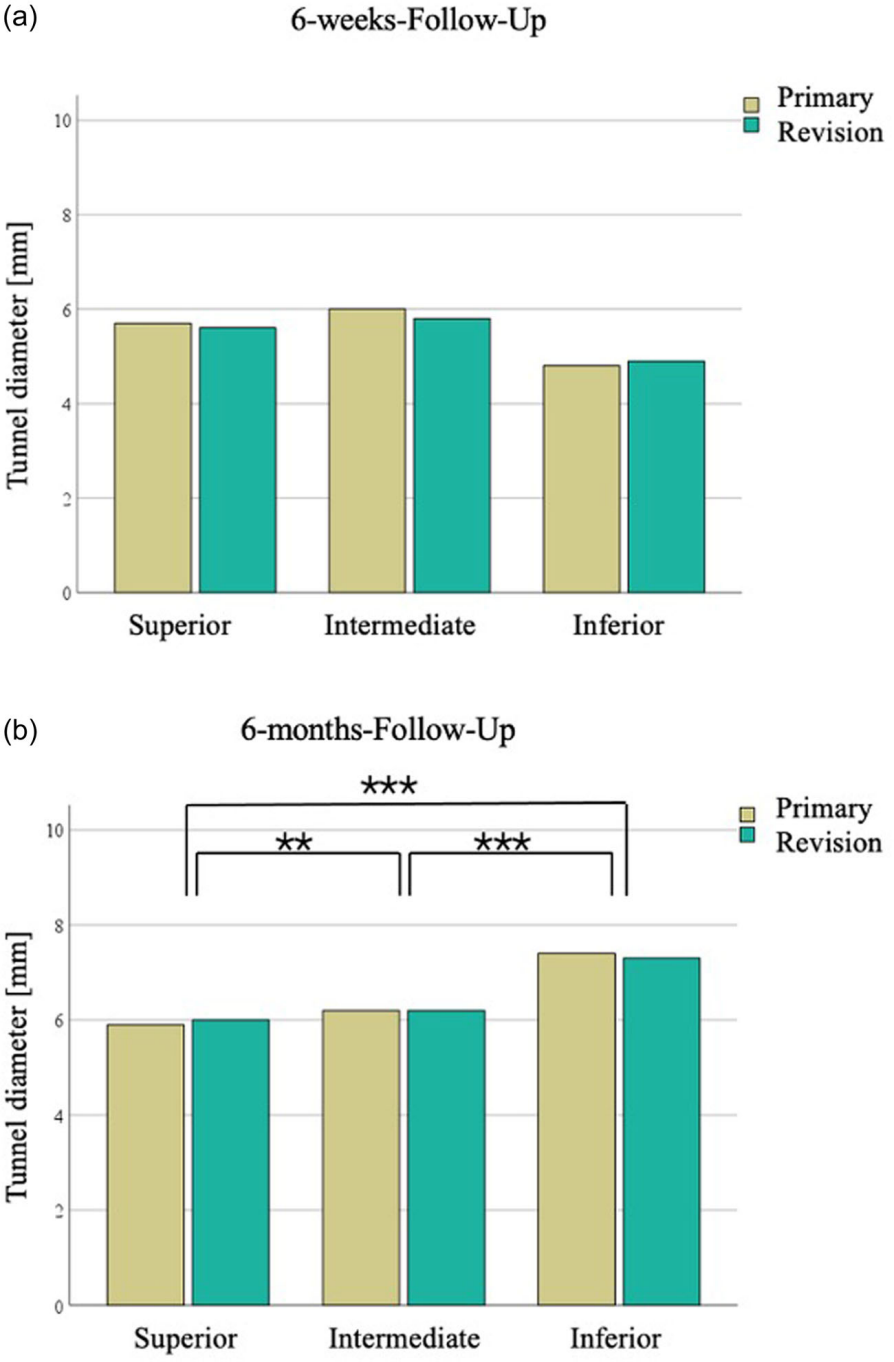
Clavicular tunnel diameter at the superior, intermediate and inferior level at the 6‐weeks‐follow‐up (a) and at the 6‐months‐follow‐up (b). **p = 0.01; ***p < 0.001.

At the 6‐weeks‐FU, the tunnel diameter was 5.7 mm (95% CI, 5.5−5.8 mm) at the superior cortex, 5.9 mm (95% CI, 5.6−6.1 mm) at the intermediate cortex and 4.9 mm (95% CI, 4.4−5.3 mm) at the inferior cortex (Figure 4a).

At the 6‐months‐FU, the tunnel diameter was sequentially larger from superior compared to intermediate (6.0 mm, 95% CI, 5.8−6.1 mm vs. 6.2 mm, 95% CI, 6.0−6.4 mm; *p* = 0.01) as well as comparing intermediate to inferior (7.3 mm, 95% CI, 7.0−7.7 mm; *p* < 0.001, respectively) or superior to inferior (*p* < 0.001), resembling an inverted V‐shape (Figure 4b).

cTW parameters were comparable across groups (Table 2).

#### Association between recurrent instability and cTW

Among the cTW parameters related to the 6‐months‐FU, the difference in cTW at the inferior cortex between the 6‐weeks‐ and 6‐months‐FU was associated with LOR (Table 3) and DPT (Table 4). Specifically, this cTW parameter was dependent on a smaller FR (*r* = −0.430; *p* = 0.032), meaning that cTW at the inferior cortex was more pronounced in cases with a greater clavicle height or a less inserted button.

**Table 3:** Correlation between loss of reduction and parameters of clavicular tunnel widening at 6‐months‐follow‐up or between follow‐ups.

Parameter compared with loss of reduction	Correlation coefficient	*p* Value
At 6‐months‐follow‐up		
Tunnel width superior clavicular level	0.132	0.530
Tunnel width intermediate clavicular level	0.024	0.909
Tunnel width inferior clavicular level	−0.098	0.642
Tunnel area	−0.106	0.615
Differences between 6‐weeks‐ and 6‐months‐follow‐up		
Tunnel width superior clavicular level	−0.012	0.955
Tunnel width intermediate clavicular level	−0.150	0.474
Tunnel width inferior clavicular level	−0.449	0.024*
Tunnel area	−0.017	0.935

* *p* < 0.05.

**Table 4:** Correlation between dynamic posterior translation and clavicular tunnel parameters at the 6‐months‐follow‐up or the difference between the 6‐weeks‐ and the 6‐months‐follow‐up.

Parameter compared with dynamic posterior translation	Correlation coefficient	*p* Value
At 6‐months‐follow‐up		
Tunnel width superior clavicular level	0.259	0.193
Tunnel width intermediate clavicular level	−0.026	0.896
Tunnel width inferior clavicular level	0.175	0.381
Tunnel area	0.287	0.146
Differences between 6‐weeks‐ and 6‐months‐follow‐up		
Tunnel width superior clavicular level	−0.082	0.696
Tunnel width intermediate clavicular level	−0.261	0.208
Tunnel width inferior clavicular level	0.421	0.036*
Tunnel area	0.206	0.324

* *p* < 0.05.

Button sintering to the isocortical level was observed in 44.4% of cases (12/27) and comparable between groups (primary: 46.7% [7/15] vs. revision: 41.7% [5/12]; *p* = 0.795).

Cases with isocortical top button sintering showed no more LOR (−3.8 mm, 95% CI, −6.3 to −1.3 mm) than cases without sintering (−3.3 mm, 95% CI, −5.7 to −0.8 mm; *p* = 0.979). They were neither associated with more DPT (11/2/2 vs. 8/3/1; *p* = 0.951). There was no subcortical sintering.

If osteolysis occurred, then only distal to the top button. It did not exceed 1 mm on each side of the button. CC ligament ossification was noted in one case.

One patient in the revision group developed a superficial wound infection that was successfully treated with oral antibiotics.

No fractures were observed.

## DISCUSSION

The main finding of this study was that arthroscopically‐assisted ACJ stabilization for chronic bidirectional ACJ instabilities exhibited similar radiologic outcomes, regardless of whether the patients had undergone previous nonoperative or surgical treatment.

A symmetric CCD was attained at the 6‐weeks‐FU, but LOR was evident at the 6‐months‐FU, along with an increased tunnel area. cTW at the singular clavicular tunnel was conical in shape, occurring increasingly towards the inferior cortex.

cTW at the inferior cortex was associated with recurrent vertical and horizontal instability and related to FR.

Managing chronic bidirectional instabilities of the ACJ poses a challenging task and necessitates individualized decision‐making. In the chronic setting, nonoperative treatment with physiotherapy should generally be performed to improve kinematics and avoid dyskinesia [7, 8].

Surgical treatment depends on injury severity. Cases with low‐grade or unidirectional ACJ instability should be considered for a sparing lateral clavicle excision or coracoacromial ligament transfer [31], while cases with high‐grade bidirectional ACJ instability should be subject to combined CC and AC stabilization [13, 15].

The current radiographic study is congruent with previous reports on the treatment of chronic ACJ instability by the use of tendon grafts [4, 5, 9, 17, 21, 25, 27, 28], which can be augmented with a synthetic device [25]. The radiologic results imply that a tendon loop configuration is not generally inferior to tunnel fixation [26].

LOR was noted between the FUs (6‐weeks and 6‐months, without differences between the primary and revision groups).

Our findings are concordant with Tauber et al. [28], who showed that, compared to an isolated CC stabilization (two tunnels), additional transacromial stabilization was associated with less LOR (3.1 vs. 6 mm) and horizontal translation. It is also concordant with Berthold et al., who found a LOR of 3.9 mm 37 months postoperatively in chronic cases that had undergone a similar CC stabilization but with AC augmentation by the graft [5]. Using a similar technique, Cerciello et al. found a LOR (4.1 mm) between directly postoperatively and the final FU of 3.8 years [9].

In a comparable study conducted by Kraus et al. [17], outcomes were derived from revision cases in a chronic context, employing dual clavicular drilling (4.5 mm) and a singular coracoid drilling (4 mm) with fixation using a transacromial tendon graft, additionally augmented synthetically (4 mm drilling). Irrespective of primary or revision surgery, they also noted cTW. However, this was only significant at the intermediate and inferior levels of the medial clavicular tunnel for the graft. Although no fractures were observed, the risk for this complication was increased by three more tunnels compared to the current study, where LOR was comparable. The current study confirms the finding that radiologic outcome remains unaffected by the type of former treatment.

The LOR may be explained by synthetic or biologic implant elongation that results from shear forces in the postoperative course, implying that the use of more stable and stiffer sutures should be evaluated.

cTW increased over time and is primarily interpreted as the result of a ‘wind‐shield wiper’ effect [6, 23] towards the inferior cortex, where the diameter was more than doubled. In contrast, the diameter at the superior and intermediate levels barely increased, leading to an inverted V‐shape.

On the one hand, this suggests that the implant interposed between the suture and bone at the upper clavicular half (FR: 0.47) contains the development of cTW. Additionally, there was no marked osteolysis adjacent to the implant device. On the other hand, the exposition of bone to the sutures at the inferior cortex was subject to cTW. These results align with findings by Bellmann et al. [3] and Berthold et al. [5]

Further analysis revealed that the increased cTW at the inferior cortex was associated with FR (although this aspect appears reserved to the LPTR implant [3]), implying that such cTW is more prominent in thicker clavicles or cases with a more superior‐seated implant device within the clavicle. The latter appears to be of less significance, although the button in this study appeared to be fully seated (approx. 5 mm insertion depth, concordant with FR and clavicle height).

In the practical sense, this association infers that cases with a thicker clavicle might benefit from a more filling button. The correlation between cTW and recurrent vertical and horizontal ACJ instability (LOR, DPT), which may be symptomatic [9, 10, 18, 21, 31], implies that an improvement in cTW could provide a clinical benefit [16, 31].

Berthold et al. reported the absence of a correlation between LOR and cTW. However, a direct comparison with our study is difficult due to the presence of two clavicular tunnels with complete tunnel coating by the implant and a singular measurement at the widest diameter of each tunnel, thereby prone to positional variability [5]. Using two tunnels suggests more limitations (and challenges) for tunnel placement in revision cases, as shown by a slightly more lateral position in our study for revision cases.

Furthermore, graft fixation by tunnel instead of loop with two clavicular tunnels instead of one inherently increases fracture risk.

Velasquez et al. [30] described risk factors for cTW after CC stabilization. Our tunnel drilling was virtually within the recommended range of 2.5−5 mm. Although dedicated ACJ ligament complex reconstruction was reportedly associated with cTW, it likely results from selection bias of a higher injury severity requiring such (additional) treatment.

Similar to Berthold et al. [5], a missing association of cTW with LOR can be explained by differences/heterogeneity in surgical treatment (specifically not being an LPTR) and measuring techniques.

### Limitations

Radiographs may be subject to projectional differences and not three‐dimensional imaging (e.g., computer tomography). A clear comparison between previous nonoperative and surgical treatment is confounded by the different techniques during index surgery (including slight alterations in mediolateral tunnel position during revision surgery) and all patients receiving prior physiotherapeutic treatment (prior physiotherapy may have provided better outcomes not related to the surgical procedure itself, and the benefit of stand‐alone physiotherapy were not assessed). Patients were rather old and female compared to typical cohorts [15, 32], with few Rockwood type II cases. These observations are, however, congruent with daily clinical practice. There was no control group, clinical parameters were not assessed, and FU was limited to 6 months (which could be the reason that no postoperative fractures were observed, especially in revision cases). Coracoid tunnel widening [14] was not assessed, and recurrent instability was reported in mm rather than severity according to Rockwood types.

Surgical treatment was performed arthroscopically‐assisted, which may be more complex in skill and cost but allows for evaluation/treatment of concomitant glenohumeral pathologies without requiring secondary implant removal [31, 32].

In general, the case number was low (and wide in age variance), which risks type‐II‐errors.

Ultimately, more research into reduced cTW, for instance by a more filling device (as indicated by Velaszquez et al. [30]), should be performed.

## CONCLUSION

Patients undergoing arthroscopically‐assisted ACJ stabilization for chronic bidirectional ACJ instabilities showed comparable radiologic results after previous nonoperative or surgical treatment.

cTW increased towards the inferior cortex and was associated with recurrent vertical and horizontal instability related to a smaller FR.

More research into reduced cTW, for instance, by a more filling device, should be performed.

## AUTHOR CONTRIBUTIONS

**Philipp Vetter**: Data curation; formal analysis; investigation; software; visualization; writing—original draft. **Frederik Bellmann**: Data curation; formal analysis; investigation; methodology; project administration; software; validation; writing—review and editing. **Larissa Eckl**: Data curation; formal analysis; investigation; methodology; project administration; software; validation; writing—review and editing. **Asimina Lazaridou**: Data curation; formal analysis; investigation; methodology; project administration; software; supervision; validation; writing—review and editing. **Markus Scheibel**: Conceptualization; data curation; investigation; methodology; project administration; resources; supervision; validation; writing—review and editing.

## CONFLICT OF INTEREST STATEMENT

The authors declare no conflict of interest.

## ETHICS STATEMENT

Ethical approval was granted by the Cantonal Ethics Commission of Zurich (BASEC‐Nr. 2021‐00675). Informed consent is granted by each included patient.

## Data Availability

Production data is available upon reasonable request.
